# Multidrug punch cards in primary care: a mixed methods study on patients' preferences and impact on adherence

**DOI:** 10.3389/fphar.2014.00220

**Published:** 2014-10-01

**Authors:** Fabienne Boeni, Kurt E. Hersberger, Isabelle Arnet

**Affiliations:** Pharmaceutical Care Research Group, Department of Pharmaceutical Sciences, University of BaselBasel, Switzerland

**Keywords:** pharmaceutical care, community pharmacy, medication adherence, primary care, dose-dispensing aids, multidrug punch card, polypharmacy, mixed methods

## Abstract

**Background:** Multidrug punch cards are frame cards with 28 plastic cavities filled with a patient's oral solid medication. They are used in primary care to facilitate medication management and to enhance adherence. Main criticism concerned handling difficulties and fading knowledge about medication of patients using them. This study aimed at exploring daily use, preferences, and adherence of primary care patients using multidrug punch cards.

**Methods:** Community pharmacies in Switzerland recruited primary care patients using multidrug punch cards. A mixed methods approach was applied with quantitative interviews performed by telephone and qualitative interviews face-to-face.

**Results:** Of 149 eligible patients from 21 community pharmacies, 22 participated 2011 in the quantitative and 11 participated 2013/14 in the qualitative interview. Patients were very satisfied with the multidrug punch cards and stated increased medication safety. All considered adherence as very important. Self-reported adherence was 10 (median) on a visual analog scale (0 = no intake, 10 = perfect adherence). The absence of package inserts and predefined handling difficulties e.g., tablets spiking at removal were not perceived as problems.

**Conclusions:** Patients are satisfied with the multidrug punch cards, feel safe, mostly have no handling problems and adhere to their treatment. Trust in health-care professionals and patients' experiences emerged as key variables for initiating multidrug punch card use and for medication adherence. This mixed methods study invalidates previous concerns about disadvantages of multidrug punch cards. Health-care professionals should actively recommend them for primary care patients with polypharmacy and poor adherence.

## Introduction

Medication management, i.e., the patient's ability to self-administrate her/his medication constitutes a major preoccupation in a patient's life (Maddigan et al., 2003; Van Dooren et al., 2010; Lecouturier et al., 2011). Physical and cognitive barriers hinder patients from removing medication from the primary and secondary packaging, from preparing it (e.g., handling a measuring cup, tablet-splitting, etc.) and from administering it the right way at the right time in the right dosage (Atkin et al., 1994; Schoberberger et al., 2007; Van Geffen et al., 2010). Medication administration errors including non-adherence and incorrect use belong to the leading causes for adverse drug reactions and related hospitalizations (Beijer and De Blaey, 2002; Gurwitz et al., 2003; Field et al., 2007; Leendertse et al., 2008). Elderly patients with polypharmacy for chronic diseases are at highest risk for such adverse drug reactions (Kongkaew et al., 2008).

The World Health Organization defined medication adherence as “the extent to which a person's behavior—taking medication, following a diet and/or executing lifestyles—corresponds with agreed recommendations from a healthcare provider” (World Health Organisation, 2003). An average of 50% of patients does not take long-term medication as prescribed (World Health Organisation, 2003), either intentionally (when the patient consciously decides not to take the medication) or unintentionally (when the patient is not able physically or cognitively to follow his own intent of taking medication as recommended). Non-adherence increases morbidity and mortality, decreases quality of life, and raises healthcare costs (Hughes et al., 2001; Ho et al., 2006, 2008; Cutler et al., 2007; Zed et al., 2008; Dragomir et al., 2010; Roebuck et al., 2011). Strategies and aids to enhance adherence have been of major interest (Bosworth et al., 2011). Dose-dispensing aids such as multidrug punch cards and pillboxes have been suggested for unintentionally non-adherent elderly patients with complex medication regimen (Cramer, 1998; Hersberger et al., 2013; Hugtenburg et al., 2013). Current literature reviews state an effect of dose-dispensing aids on adherence and clinical outcomes, but robust and reproducible studies are lacking (Mahtani et al., 2011; Zedler et al., 2011; Boeni et al., 2014).

Several studies have described handling difficulties with the use of dose-dispensing aids (Macdonald et al., 1977; Gould et al., 2009; Nunney et al., 2011; Adams et al., 2013). In one study, six out of fifteen patients put the loose tablets from a dose-dispensing aid back into a bottle because they could not handle the device (Macdonald et al., 1977). Another study reported that patients who elaborated their own medication management system tended to return to it after initiation of a prefilled dose-dispensing aid (Nunney et al., 2011). Such misuse is critical for patient safety. Medication knowledge has been advocated as essential for patient safety. Often, prepackaged dose-dispensing aids are delivered directly to the patient's home and thus were observed to reduce contact between the pharmacist and the patient. In connection, knowledge about self-administered medication seemed to be poorer in patients with dose-dispensing aids than in patients who manage their medication on their own (Nunney and Raynor, 2001; Kwint et al., 2013). A recommendation paper of the Royal Pharmaceutical Society criticizes the distribution of dose-dispensing aids to all patients without assessing their capabilities and needs (Royal Pharmaceutical Society, 2013). In Switzerland, one single criterion (intake of >3 different medications) is required by the health insurance to supply reimbursed dose-dispensing service (repackaging of solid oral medication into dose-dispensing aids by a healthcare provider) by the community pharmacy to primary care patients.

Two qualitative studies explored the views of patients using various dose-dispensing aids (Lecouturier et al., 2011; Nunney et al., 2011). Findings of these studies indicated that one group of patients saw clear benefits in dose-dispensing aids, whereas the other group felt patronized and restricted in liberty. Some of the patients had handling problems with the devices and troubles with identifying their medication. Both studies concluded that future studies have to clarify which patients benefit most from dose-dispensing aids and how to recognize them in primary care.

**Figure 1:**
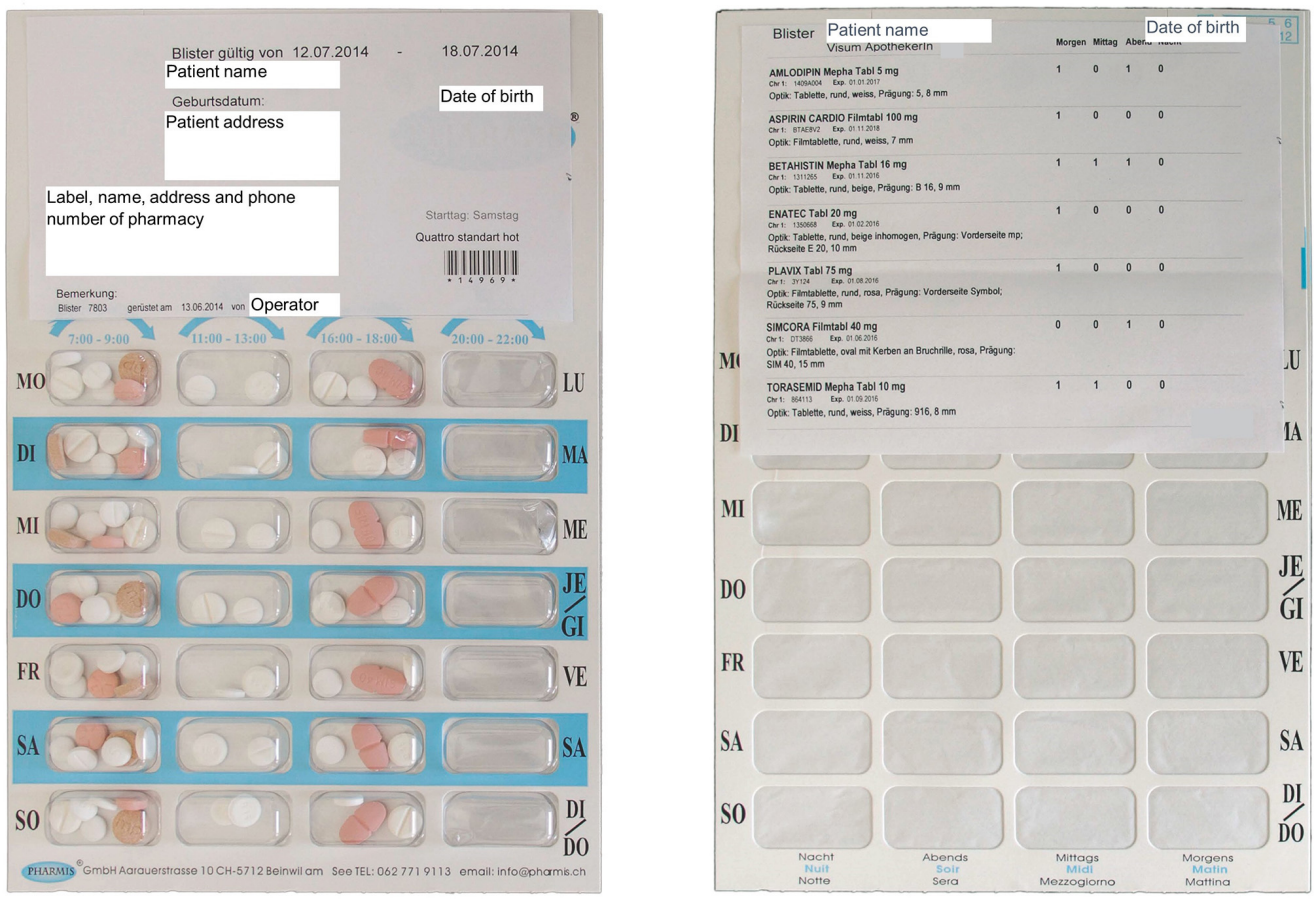
**Multidrug punch card**. Front side (**left**): 28 plastic cavities with visible packaged medication and labeling with patient and pharmacy information. Back side (**right**): 28 cavities sealed with foil and marked with indication of dosing time (morning, lunch, evening, night; Monday–Friday); the adhesive medication plan labels brand name, dose, administration number, dosing frequency, size, color, imprint, batch number, and expiration date of each packaged drug. All specifications are in German.

Multidrug punch cards are disposable frame cards with plastic cavities, sealed with a foil backing, with typically 28 compartments, filled by pharmacy staff, by a specialized company or an automated system (Figure 1). They provide a visual reminder for medication intake, the possibility of adherence self-monitoring and the saving of time, costs, healthcare resources (e.g., home care nursing), and medication waste. Multidrug punch cards were introduced in Switzerland in 2002 together with a documentation software for community pharmacies (Pharmis GmbH).

We conducted a mixed methods study to assess experiences, attitudes, and adherence of primary care patients using multidrug punch cards in Switzerland. We aimed at investigating the preferences of primary care patients using multidrug punch cards in daily life, at compiling a profile of the primary care patient benefitting most of the multidrug punch cards' use and thus at facilitating a targeted adherence interventions. The results should advance the rational distribution of multidrug punch cards and connected healthcare services.

## Materials and methods

Quantitative interviews were performed in 2011 and qualitative interviews were conducted sequentially in 2013/2014 to clarify the results. A positive notification was obtained by the regional ethic boards. Good Reporting of A Mixed Methods Study (GRAMMS) guidelines were considered (O'Cathain et al., 2008).

### Recruitment and inclusion criteria

In 2011, all community pharmacies in the cantons of Basel-Stadt, Baselland, Aargau, and Solothurn (Switzerland) delivering multidrug punch cards to primary care patients were asked to participate in the recruitment of patients for the quantitative interviews. Community pharmacies providing multidrug punch cards to primary care patients in the cantons of Basel-Stadt and Baselland were re-invited in 2013/2014 to recruit patients for the qualitative interview. One pharmacist per pharmacy was instructed for recruitment. Patients were eligible if they had used multidrug punch cards for at least 3 months, lived independently, administering medications without external help, spoke German and were able to give informed consent. The pharmacists decided upon eligibility of the patients and recruited them by phone or face-to-face at their next visit at the pharmacy. The study team received the contact details of accepting patients and called them to fix a date for the interview. Patient information and the informed consent form were provided through the pharmacy or at the interview. For both interviews, patients were approached in the same manner, irrespective of participation in the first, quantitative interview.

### Instruments

A quantitative questionnaire was developed containing five domains (living situation, general questions about the multidrug punch cards, handling, design, and medication adherence). Answers were indicated as multiple choice, Lickert-scales, on visual analog scales (VAS) or were open ended. The questionnaire was validated for feasibility, understandability and consistency of the scales. The questionnaire comprised 31 questions and took 30 min to conduct. Demographic parameters included age, sex, living situation, education, status of employment, and number of medications. Adherence was measured through patient self-report on a VAS ranging from 0 (taking no medication) to 10 (taking all prescribed medication every day at the right time). The term “medication adherence” is not colloquially used in Swiss German. We therefore replaced it with “fidelity to therapy” [Therapietreue], which we suggested to be more understandable. The term was explained to the patients before patient self-report of adherence by the VAS and discussion about importance of adherence was conducted.

A qualitative topic guide was constructed upon the results of the quantitative questionnaire with themes that remained unclear or contradictory. The topic guide and the course of the interview were piloted with two patients who were not included into the final analysis. Adoption of the multidrug punch cards, acceptance, use in everyday life, design, and medication adherence built the five domains. Subtopics were outlined with 19 pre-worded questions. Demographics and adherence were asked in the same manner as in the quantitative interview. Both, the quantitative questionnaire and the qualitative topic guide were applied as interviews. After interviews had been held, the current medication plan was obtained from the corresponding pharmacies.

The quantitative interview was conducted by telephone after informed consent was received by post. One interviewer performed the interview, reading out the questions and the possible answers of the questionnaire. Immediate feedback was requested from the interviewed patient for assurance of ticking the right box.

The qualitative interview was held face-to-face at the patient's home or at the pharmacy in a separate room. FB led the interview and another researcher asked in-depth questions. Each domain was introduced to the patients by a general open-ended question to allow the patients to answer freely. Subtopics that remained untouched were then explored by further questions. The order of the domains and questions followed the patient's answers. The interviews were held in Swiss German and were audiotaped. One research assistant (NR) orthographically transcribed the recordings in German language, preserving dialect expressions. All transcriptions were double-checked by FB.

### Analysis

The quantitative interviews were analyzed descriptively by using Microsoft Excel 2013 for Windows (Microsoft Corporation, Redmond, WA, USA). Answers to open questions were categorized and analyzed quantitatively. Missing data were excluded from the analysis. Numbers of valid answers are given for each question.

Transcriptions of qualitative interviews were transferred to MAXQDA V. 11 for Windows (VERBI GmbH, Berlin, Germany). Data were analyzed analogously to a five-stage “framework approach” developed for applied qualitative research (Pope et al., 2000; Lecouturier et al., 2011). A coding framework was constituted by preliminary coding of five interviews. Domains related to the original topics were structured as main codes and emergent themes formed sub-codes. After verification, the coding framework was applied to all interviews. Coding was performed manually line-by-line by FB. Codes of all interviews were grouped for detection of associations and patterns. Quotations were selected to illustrate the analysis. They were translated into English by FB and checked by a native English speaker. Original German transcriptions of the quotations are listed in the Supplementary Material.

Quantitative and qualitative data are presented in direct relation to each other in the Results' section and were integrated by FB on the level of interpretation. Qualitative data were used to complete and explain findings from the quantitative interviews.

## Results

### Demographics

In 2011, 33 of 266 community pharmacies in the cantons of Basel-Stadt, Baselland, Aargau, and Solothurn delivered multidrug punch cards, mainly to nursing home patients. Of the 25 pharmacies supplying primary care patients, 21 participated in the recruitment of the patients for the quantitative interview. They supplied a total of *n*_quant_ = 149 patients, of whom 25 (17%) were contacted by the study team and 22 (15%) consented to perform the quantitative interview.

In 2013/2014, 13 of 124 community pharmacies in the cantons of Basel-Stadt and Baselland supplied primary care patients with multidrug punch cards and 6 participated in the recruitment of the patients for the qualitative interviews. Of a total of *n*_qual_ = 60 patients, 18 (30%) were recruited and 16 (27%) consented to perform the qualitative interviews. Five patients had to be excluded from the analysis, two because they participated in the pilot study, two because of language difficulties and one because of the use of a dose-dispensing aid other than multidrug punch cards. Reasons for exclusion by the pharmacist for the quantitative and qualitative interviews were (*n*_quant_/*n*_qual_): cognitive or psychological barrier 30/16; participation rejected 27/13; home care 25/4; language barrier 19/11; patient unreachable 6/6; multidrug punch card use for less than 3 months 6/3; terminal medical condition 2/0; deceased 2/2; multidrug punch cards abandoned 1/0; reason unknown 6/3. Patient demographics are listed in Table 1. Mean durations of the quantitative and qualitative interviews were 28.5 (SD ± 7.5) and 42.8 (SD ± 14.2) min, respectively.

**Table 1:** **Demographics of patients participating in the quantitative and in the qualitative interviews**.

**Patient demographics**		**Quantitative interview**	**Qualitative interview**
Participants, n		22	11
Age, median (range) [years]		71 (37–96)	76 (27–91)
Sex, n	Female	14	5
	Male	8	6
Living situation, n	Alone	13	10
	With partner	9	1
Education, n	No school graduation	2	2
	Primary school	19	8
	University	1	1
Status of employment, n	Employed Retired/unemployed	1	0
		21	11
Numbers of medications, median (range)	In multidrug punch cards	7 (4–13)	7 (4–12)
	Additional (outside multidrug punch cards)	1 (0–4)	1 (0–3)

### Reason to recommend multidrug punch cards

According to the quantitative interviews, multidrug punch cards were recommended by pharmacists in 54% of the cases, by physicians in 18%, by relatives in 14%, and by others in 14%. Of the 16 patients who had the multidrug punch cards recommended by a pharmacist or a physician, 14 remembered one or several reasons: (new) prescription of numerous medications and/or complex regimen (*n* = 7), facilitation of medication management (*n* = 6), poor adherence (*n* = 6), hospital discharge (*n* = 3), and medication abuse (*n* = 2).

Qualitative interviews largely confirmed these reasons. The medical condition was named as principal reason which finally resulted in getting multidrug punch cards (*n* = 4). The same four patients, who stated that they were confused with their medication or had difficulties in handling it, also declared that non-adherence was a reason for the recommendation of the multidrug punch cards. Difficulty/confusion: *“I always have messy cupboards xxx. I've always had a box with one pill here, one pill there. Packaged like this [in regular packaging], right? Then I just did “tschak, tschak, tschak” back and forth. And in time it seemed to me, it's not the best solution, is it.”* (P7) [xxx = garbled speech, unable to make an educated guess]. Non-adherence: *“Sometimes it's also happened that I've forgotten one [tablet] or so.”* (P7). These patients mentioned their problems in the community pharmacy or to a relative, which led to the recommendation of multidrug punch cards. Four patients received the multidrug punch cards on prescription or by arrangement between the general physician (GP) and the pharmacist. Two of them did not remember having talked about it to the GP or the pharmacist prior to the initiation of the multidrug punch cards. One patient explained that it was his own idea to save money, because the size of packages often did not fit his needs. The packaging was proposed as solution by the GP. *“[…] either they [the pharmacy] make packs with only 10 [tablets], and then this doesn't really go far. Or they [the pharmacy] make a pack with 50 or 100 [tablets] and I don't need them either. And then, there's a lot lost. And that way [with the multidrug punch cards], I really have only the medication that I need.”* (P2).

### Advantages and disadvantages of multidrug punch cards

In the quantitative interviews, all 22 patients felt well cared for by the pharmacy. All were satisfied with the multidrug punch cards, 20 of them very much. Facilitation of medication management and the reminding of medication intake were the main advantages mentioned. Overall, 67 advantages and 12 disadvantages were named (Table 2). Twenty patients liked the design of the multidrug punch cards and agreed fully that it was clearly arranged. The orientation according to the written dosing times was judged as very easy by 21 patients and as easy by one. However, the patients stated uniformly that the functionality was more important than the design. The multidrug punch cards were rated as practical and very robust by all 22 patients.

**Table 2:** **Advantages and disadvantages named by all 22 patients of the quantitative interview in an open-ended question**.

**Advantages**		**Disadvantages**	
Facilitation of medication management	22	Difficult medication removal	5
Reminder for medication intake	14	Missing package insert	3
Clear design	7	High refill frequency	2
Control	6	Waste	1
Medication safety	4	Missing confidentiality	1
Organization	4		
Communication	2		
Facilitation of therapy adjustment	2		
Mentioned once: recycling of medication, space-saving, hygiene, documentation, home delivery, rationing	6		
Total	67		12

The satisfaction was also high in the qualitative interviews with 55 passages coded with positive expressions about the multidrug punch cards [e.g., *“This is marvelous!”* (P1)]. There were no corresponding negative remarks. Most patients said that they much preferred the multidrug punch cards to their prior medication management system. It was a facilitation, not only for medication management, but also for their life: it was less time consuming, they did not have to reflect which “box” to use at which dosing times and they did not have to store numerous medication boxes. *“This [multidrug punch cards] really simplifies my life!”* (P1). *“Again, one concern less for me!”* (P5). Patients also highlighted the clarity and order of the multidrug punch cards. The layout helped them to orientate themselves. Interviewer: *“And why do you like it, when it [the medication] is packaged like this [in the multidrug punch cards]?”—Patient: “You have an overview. […]”* (P8). Few comments concerned the high-level hygiene and the suitability for old and/or forgetful people. Only four negative comments were issued by three patients: the sound of the multidrug punch cards while handling was displeasing, a long sheet with the medication plan glued on the back was unpractical while removing medication, the assumption that the handling could be difficult for people with disabilities and the lack of package insert and information to identify the tablets. *“The disadvantage, I find a bit is that you don't have an overview of the tablets. Now, I really can't…. Where there is a heart on it [the tablet], I know it is for the heart somehow, but on the whole, I do not know what I here [take]…. Well, everything is written in the back, isn't it, for me. I don't know if they do that in general or not?”* (P4). This comment was stated by a patient who also criticized that he could not understand the information of the medication plan glued on the back, he thought it was written in Latin. On the other hand, the lack of package insert did not trouble other patients and was appreciated as an advantage by several patients.

### Handling of the multidrug punch cards

In the quantitative interviews, 21 out of 22 patients were very satisfied with the handling of the multidrug punch cards. Nineteen patients pushed the medication out with their fingers. Of five patients cutting the foil on the backside, four seldom or never had trouble in pushing out the tablets. In total, 14 (64%) patients indicated never having trouble with removing medication from the multidrug punch cards. Eight patients had technical or physical difficulties: tablets spiked at removal (*n* = 5); tablets stuck in the cavity at removal (*n* = 4); dexterity problems (*n* = 3); cavity too fully loaded (*n* = 1).

During the qualitative interviews, patients were asked to demonstrate with a demo multidrug punch card how they removed their medication. All 11 patients removed the mock medication without trouble, but sometimes it spiked. Although some patients admitted that this happened from time to time with their own multidrug punch cards too, they mostly did not see it as a drawback. Some of the patients described problems with removing medications at the very beginning of multidrug punch card use, but they developed their own strategy to overcome these problems. Most patients had not been instructed how to use the multidrug punch cards or did not remember it. They negated the need for it, because they found the multidrug punch cards self-explaining. Four patients reported that they daily removed the content of the cavities in advance into a separate little box or bowl. This was practical to them because they kept the medication ready and could not mix it up, or they had it in their pocket in case they left home. One patient was sure that she would forget the intake in the morning, if she did not prepare the dose the evening before. *“Because I have to prepare them, otherwise I would really…, I have to tell you honestly, I would forget them [the medication].”* (P8). Two patients told that they manipulated the multidrug punch cards for their purpose. The main motivation was cutting the size for storage or transport. *“[…]. If I know, of course, I will leave for three days, then I cut it [multidrug punch card] here.”* (P2). One patient also pushed medication into the cavities or took some of the filled medication out if there was a short-term change in medication therapy. She did not report these therapy changes to the pharmacy until she was sure it was fixed. *“[…]. And after this, just once for this evening I did it, so that I don't have to mess around for a long time, I took the two [tablets] that I have to take anyway, I pushed them in here and the blue one I already pushed out [of the multidrug punch card]. That's how I work with the blister [= multidrug punch card].”* (P1).

### Safety issues

In the quantitative interviews, safety and control were named by four and six patients, respectively, as an advantage of the multidrug punch cards (Table 2). All 22 patients stated that they felt safer in medication management with the multidrug punch cards than without. All patients agreed fully that they could read the text with the information written and glued on the back of the multidrug punch cards without problems. Three patients admitted that they never read this text. Three patients named the missing package insert as a disadvantage.

**Figure 2:**
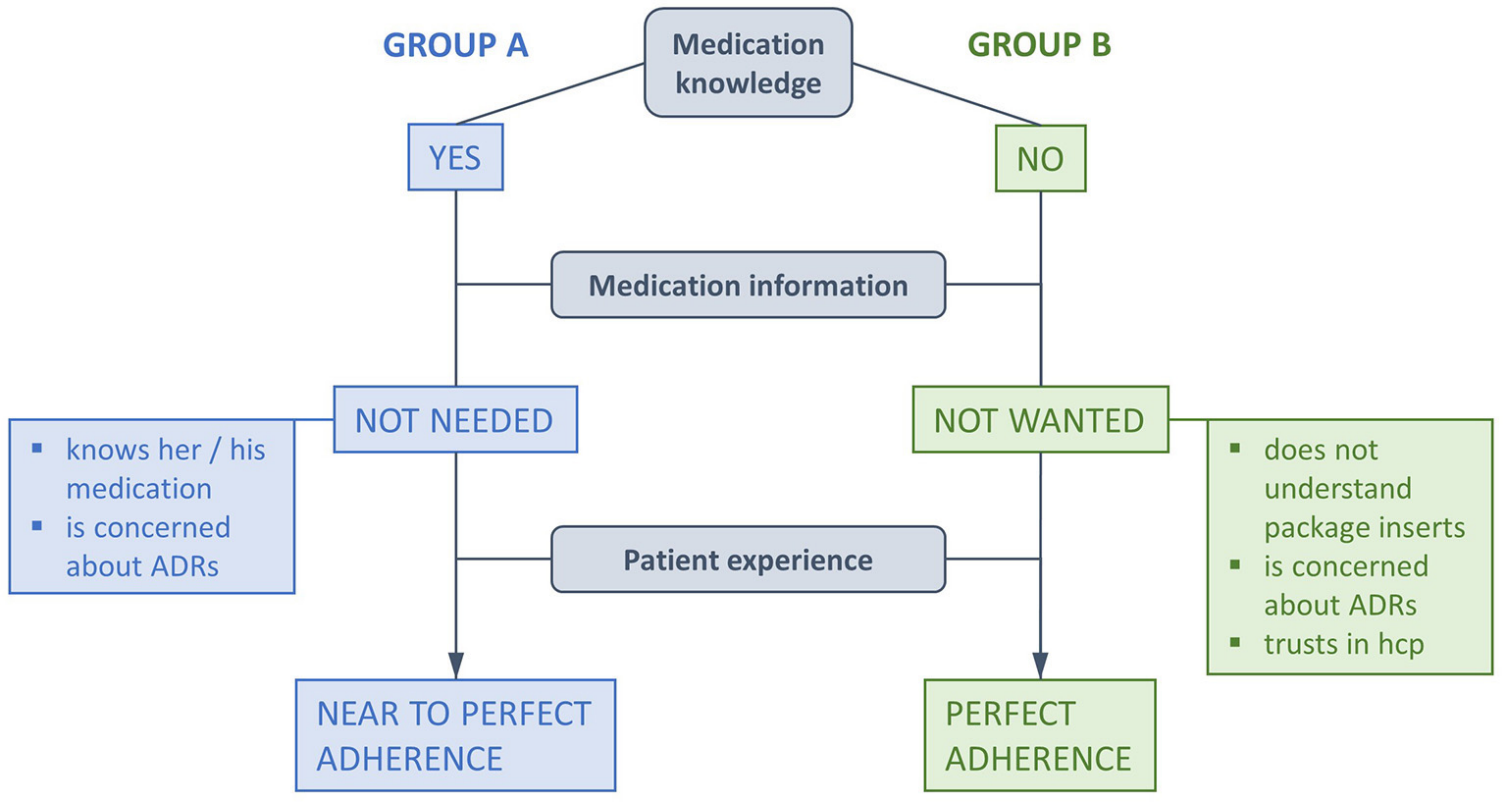
**Adherence elements emerging from qualitative interviews**. Although all patient stressed perfect adherence, statements of Group A allowed margins for time of medication intake (= near to perfect adherence). “Medication knowledge” relates to a patients' confidence to appoint the name and/or the indication of the medication and/or to identify the tablets (ADRs, adverse drug reactions; hcp, health-care professionals).

The topic safety was explored in-depth in the qualitative interviews. All 11 patients confirmed that the multidrug punch cards made them feel safe in managing medication. The main reasons were the overview of their medication and to be in control of medication intake. It was very important for them to be sure they had the right medication at the right time. *“Yes, I would say there is a kind of safety in it [multidrug punch cards]. Then I'm sure I took the right one, here.”* (P2). Some patients mentioned in that context that they believed the medication filling to be correct and that they could rely on the controls of the health-care professionals. Nevertheless, all 11 patients reported that they controlled the tablets immediately after removal by number, shape or color. Two patients felt safe because the medication was incorporated in a package that was hygienic and robust. In relation to medication knowledge, the patients could be divided in two groups (Figure 2). Group A was confident to know the name and indication of their medication, could more or less identify the tablets in the multidrug punch cards and stated that they did not need further information or a package insert. Group A/Knowledge: *“I know exactly what I have to take, […].”* (P5). Group A/Package insert: *“Because if I have to read the package insert, either I have to or I want to, I suffer from everything that is written there. And I don't want that at all.”* (P1). Group B did not know the name and indication of their medication and could mostly not identify the tablets. All patients of Group B except one did not want more information because they declared not to understand it. The package insert was refused quite fiercely by some patients and was named as a reason for denied medication intake. All patients of group B explained that they were faithful to the pharmacy for years and that they trusted health-care professionals. Trust and fidelity to the pharmacy also coincided with statements of perfect medication adherence. Group B/Knowledge: Interviewer: “*How well do you know which tablet is which, for example?” Patient: “I don't know.”* (P9). Group B/Package insert: Patient: *“But what the other one is, I don't know.”— Interviewer: “You don't know it. Would you like to know it then? So, do you mind not knowing it?”—Patient: “Well, I don't know if I would actually like to know it or not.”—Interviewer: “That means this fits for you then?”—Patient: “You know, this would… if, if this was something that… This would concern me very much. […]”* (P8). Group B/Package insert/trust and fidelity: *“[…]. I trust you and the physicians. I'm not interested in this because I don't understand it anyway. What's in it and what's written on it [in/on the multidrug punch card] and so. No, I never look at it.”* (P3). The medication plan glued on the backside of the multidrug punch cards was very much appreciated and was declared to contain enough information about the medication and the user. Some patients saw it as a major advantage in safety because they could give the correct names and dosages of their medication to physicians at first consultation or at admission to the hospital. Two patients told that they requested oral and written information on medication from the pharmacy if they had specific questions. All 11 patients described their contact to the pharmacy to be very good and the pharmacy team to be very friendly.

### Adherence

In the quantitative interviews, patients indicated that the multidrug punch cards were a tool to remind them of medication intake. Compared to their prior medication management system, 21 patients rated a relative improvement of +37% (*SD* ± 43%) for taking adherence and 19 patients rated a relative improvement of +38% (SD ± 43%) for timing adherence after the initiation of multidrug punch cards. One patient estimated his timing adherence to be 13% worse with the multidrug punch cards than without. Median self-reported adherence of the 33 patients participating in quantitative and qualitative interviews was 10 on the VAS (range 3–10).

In the qualitative interviews, all 11 patients stated that they did not know the term adherence [fidelity to therapy, Therapietreue], but three patients could imagine the rough sense of it. *“Yes, I stick to the rules. Which I get ordered, now about the therapy, sort of… yes. I do what I should and not…. Faithful to therapy, like this. Xxx. If you now get medication to calm down, if you… have a fit. Then I would say, fidelity to therapy is really if you just take it at the right moment.”* (P6). Others related it to physical therapy because in Swiss German the term “therapy” [Therapie] theoretically stands for various kinds of therapy, but is colloquially often used for physical therapy. Some patients had no idea of the meaning of the term “fidelity to therapy.” Two patients remembered that they had a talk with their health-care professionals about adherence, but the majority thought this was self-evident and that they did not need further explanations. All 11 patients declared that medication adherence was very important for them and emphasized their willingness to be adherent. As reasons they indicated that it made sense to follow the physician's directions, that they would benefit from the therapy and that they would suffer from medical consequences if they were non-adherent. One patient even stated that pharmacotherapy was existential for her. All patients who feared medical consequences of non-adherence had a history of an adverse medical event (e.g., heart attack) or suffered from a medical condition, which they had to keep under strict control (e.g., diabetes mellitus, epilepsy). *“I know it [the medication] holds off a lot, when you had two heart attacks, then you know what it means to take medication. Then you really take it [the medication].”* (P9). *“Well, what do I want? There is nothing else for it. It only benefits me, if I take it, right? I don't want to sit in the hospital again.”* (P8). Statements about adherence matched Groups A and B defined in the Safety's section. Patients from Group A were more liberal and reported that they were fine with a margin for time of medication intake. Patients from Group B were anxious about leaving out one tablet or taking one dose too late since they were sure to sense immediate consequences. *“If I did not take them, I would feel it. So, I would have to go soon, most likely…, so maybe the second day at most [after missing a tablet], I would already have to go to the physician and say: ‘I don’t feel well anymore'. So yea, I would feel it.”* (P10). Three patients believed that they would forget medication intake if they had to prepare the medication themselves out of the regular packaging, if the multidrug punch card was stored at a hidden place, or if there were more dosing times. Most patients who admitted that they forgot medication intake with their prior medication management system also forgot intake with the use of the multidrug punch cards, but much less than before. The visualization of the doses would reveal their omission and allow them to make up the intake. One patient had problems remembering the short-term therapy that she was managing besides the multidrug punch cards. Three patients were absolutely sure that they never forgot medication intake. Strategies to remember medication included defining an eye-catching place of storage for four patients, setting an alarm for two patients and embedding medication taking into a ritual or daily routine for four patients. All four patients who had defined a special place of storage reported always seeing it and therefore remembering medication intake. Patients who had the medication intake embedded in their daily routine told that they did not have to remember medication taking as a separate action, it was more like an automatism within their normal activities. They also did not need to control the multidrug punch cards to ensure timely intake. *“It is, it's like automatic, right? When I'm sitting, having my breakfast at the table, then I just do it and then it's done. And then I put it [the medication] into the plate and the matter is settled.”* (P5). *“I always take all of them. I always take them how I have to, I don't have to control it.”* (P9). For most patients control of intake was an additional step of safety. *“But here [with the multidrug punch card] you have control after all! Here you have it, you are sure that you took the right thing [medication].”* (P11). *“I see it at first sight. I had it, I took it, I know it.”* (P2).

## Discussion

We combined quantitative and qualitative methods in an explanatory way to investigate the profile of multidrug punch card users in-depth, and the influence of the dose-dispensing aid on their adherence. Our primary care patient using multidrug punch cards reports high level of satisfaction with the multidrug punch cards, few handling difficulties and high medication safety. She/he declares currently highest medication adherence and improved adherence compared to her/his prior medication management. Our results support the assumption that unintentionally non-adherent patients represent a target population for dose dispensing aids (Cramer, 1998; Hugtenburg et al., 2013) and highlight some key variables which health-care professionals may assess while recommending multidrug punch cards to patients with polypharmacy.

The typical independent primary care patient accepting to use multidrug punch cards is over 70 years old, has a low education grade, is retired, lives alone, favors tidiness, rituals, and daily routines and is unable or reluctant to leave home. She/he trusts the health-care professionals, is a regular customer of the same community pharmacy, is motivated to conduct a healthy life and has a feeling of high necessity for medication. The association of adherence with the necessity for medication intake is well-known and has been used as an integral part of the “believes about medicines questionnaire,” an instrument to assess adherence (Horne and Weinman, 1999).

Our patients much preferred the multidrug punch cards to their prior medication management and reported improved adherence of even +37% after the initiation of the device. Significantly increased adherence was also demonstrated by five out of six randomized controlled trials investigating the use of multidrug punch cards in primary care patients (Crome et al., 1982; Ware et al., 1991; Lee et al., 2006; Schneider et al., 2008; Valenstein et al., 2009). Additionally, in two of these studies (Lee et al., 2006; Schneider et al., 2008) cardiovascular patients with polypharmacy achieved significantly improved clinical outcomes (e.g., blood pressure, LDL cholesterol). Thus, major improvement of adherence and of associated outcomes by the use of multidrug punch cards are likely.

In our study, patients claimed their perfectly adherent behavior to be motivated by a personal experience of benefit if they adhered to the physician's orders or by a fear of medical consequences if they did not. These findings correspond to the role of patients' experiences denoted as crucial for clinical safety and effectiveness (Doyle et al., 2013). Trust toward the pharmacy emerged also as a reason for high adherence, since the participants expressing trust toward health-care professionals most explicitly, were most accurate with their medication plan. This attitude is characterized as the “passive medication user,” representing one out of three different types of medication intake-behavior (Dowell and Hudson, 1997). We thus suggest that the population of “passive medication users” could be a target group for the use of multidrug punch cards. If we add that high fidelity to the pharmacy is associated with increased medication adherence and decreased adverse drug reactions (Marcum et al., 2014), we can suppose that multiple key variables at different levels permit to reach a perfect medication intake behavior (trust in the institution/health-care professionals; perceived benefits of the management system; fear of negative consequences) (Figure 2).

Although multidrug punch cards do not feature an explicit reminder function, its storage at a strategic visible place helped the patients to remember medication intake. In particular, it allowed immediate visual control of the intakes, the performed ones as well as the forgotten ones. An advanced strategy seems the integration of the medication intake into daily routine to become an “automatism;” the patients even did not have to think about medication intake. Habits and routines have long been described to be beneficial for general adherence (Reach, 2005; Kripalani et al., 2010) as well as for dose-dispensing aids (Lecouturier et al., 2011). As a consequence, recommending multidrug punch cards should include an assessment of the patients' daily habits and routines.

Reasons for recommendation of multidrug punch cards and major advantages assessed in our study e.g., facilitation of medication therapy and improvement of adherence, mostly coincided with results of two qualitative studies on primary care patients using different types of dose-dispensing aids (e.g., pillboxes, multidrug punch cards, etc.) (Lecouturier et al., 2011; Nunney et al., 2011).

Absence of medication information—due to the dispensing of multidrug punch cards without package inserts—was of minor importance in the quantitative interviews. The in-depth exploration of the qualitative interviews confirmed that the patients were satisfied with a minimum of medication information. Only two patients requested written or oral medication information from the pharmacy. These findings might appear controversial, since a lack of medication information has been related to a reduction of knowledge resulting in a dangerous loss of skills and autonomy of the patient (Nunney and Raynor, 2001; Nunney et al., 2011; Royal Pharmaceutical Society, 2013). Inversely, good medication knowledge was suggested to reduce inappropriate medication administration, adverse events and non-adherence, and hence to increase medication safety (Kim et al., 2006; Field et al., 2007; Pernod et al., 2008; Chan et al., 2013). However, these investigations were not performed within a population using multidrug punch cards. Since their use spares the handling of regular packaged medication, a different type of knowledge seems needed by those patients than the information contained in package inserts. Our assumptions are strengthened by a recent study showing that patients over 65 years with dose-dispensing aids were significantly more adherent (*n* = 119) but less knowledgeable than patients who managed their medication by themselves (*n* = 96) (Kwint et al., 2013). Finally, since multidrug punch cards *per se* reduce potential errors of administration to a minimum, a relation to medication knowledge is unlikely.

Handling problems (e.g., difficulty in removing medication, confusing inscriptions when to take the medication, etc.) were claimed to constitute a major reason for reduced medication safety with dose-dispensing aids (Macdonald et al., 1977; Gould et al., 2009; Nunney et al., 2011). Consequently, the small number of handling problems in the quantitative interviews was surprising. However, the qualitative interviews confirmed the first findings and revealed a major contribution of multidrug punch cards to the patients' feeling of medication safety. The clear design of the multidrug punch cards assured its safe use. Hence, for most patients instruction was dispensable.

For practice, our study implies that medication management and non-adherence should be addressed actively through health-care professionals. The profiling enables selecting the right patients, provides arguments for recommendation and points out relevant issues for advancement of dose-dispensing service. Initially, trust between the patient and the health-care professional has to be established and patients' experiences and habits should be included into adherence counseling. While recommending multidrug punch cards, pharmacists should emphasize the facilitation of medication management and the increased medication safety. Based on our results, other strategies to advance dose-dispensing service and increase safety might be considered e.g., regular medication review of the packaged medication by a pharmacist (Kwint et al., 2011), giving instruction on multidrug punch cards if necessary (anticipation of handling difficulties, integration into life-style, reminder strategies), inclusion of short-term medication into the packaging, detailed instruction of separate medication, and regular contact between pharmacy and patient.

### Strengths and limitations

The strength of this study was the deeper explanation of ambiguous quantitative data through qualitative interviews. To our knowledge, this is the first study with a mixed methods approach in the field of dose-dispensing aids and their impact on medication adherence.

Our study results are limited through several points. First, our study sample is small. On one hand, this is due to the effective small number of primary care patients, who are using multidrug punch cards without external help. In Switzerland, multidrug punch cards were originally intended for the supply of nursing homes. Only in the last few years, they were recommended to primary care patients. Further, the primary care patients selected by the pharmacists as multidrug punch card users really were the target group for this type of adherence aid (cognitive or psychological barrier, home care, language barrier), but turned out to be inadequate for our study. On the other hand, about half of the adequate patients refused study participation. Telephone interviews constituted a major barrier for recruitment. Conducting interviews at home or at the pharmacy were more acceptable. Second, the high level of satisfaction may reflect a selection bias. We can assume that patients unsatisfied with the multidrug punch cards might not have been willing to consent for interviews, especially if invited by the provider of the unsatisfactory device. Further, the recruiting pharmacist may have approached satisfied users among her/his patients to take part in the study. The problem-free handling of the multidrug punch cards that we observed might be the result of a further selection bias, since we excluded cognitively impaired patients who are known to experience difficulties with the handling of any medication packaging (Atkin et al., 1994; Adams et al., 2013). Additionally, because our participants had to use the punch cards at least 3 months for inclusion, initially encountered difficulties may have been solved already. Third, reporting and interviewer biases may have interfered with study results. Since there were no differences observed by location of interview, the conduction of the interviews at the pharmacy does not seem to have influenced the patients' answers. Fourth, adherence was measured through patient self-report which has been described not to be fully reliable and often overestimated (Zeller et al., 2008). However, the conformity with similar studies (Lee et al., 2006; Kwint et al., 2013) endorses our results. Fifth, this study represents the views of patients solely using multidrug punch cards and cannot be generalized to patients using other dose-dispensing aids.

### Outlook

Future research should aim at developing studies with larger populations to enable generalization. The development of an assessment tool for non-adherent patients to provide targeted interventions should be a priority. Clarification of the impact of multidrug punch cards on patient-oriented outcomes should be aspired. Younger patients with complex medication regimen should be interviewed about their preferences to clarify the benefit of multidrug punch cards for additional populations.

## Conclusions

Characteristics of primary care patients using multidrug punch cards include age over 70 years, low education grade, living alone, appreciation for tidiness and daily routine, trust in health-care professionals, fidelity to pharmacy, and motivation for a healthy lifestyle and medication adherence. The patients are satisfied with the multidrug punch cards, feel safe, mostly have no handling problems and adhere perfectly to their treatment. Multidrug punch cards constitute a simplification for their lives, offer a clear overview of hygienically packaged medication, a reminder function and a possibility for adherence monitoring. Key variables for initiating multidrug punch card use and for medication adherence are trust in health-care professionals and patients' experiences. This mixed methods study attenuates previous concerns about disadvantages of multidrug punch cards. Hence, health-care professionals should actively recommend them for primary care patients with polypharmacy and poor adherence.

## Author contributions

All authors conceptualized the study. Fabienne Boeni conducted the interviews, analyzed data and drafted the manuscript. Kurt E. Hersberger and Isabelle Arnet reviewed the draft critically for intellectual content.

### Conflict of interest statement

The authors declare that the research was conducted in the absence of any commercial or financial relationships that could be construed as a potential conflict of interest.
