# Supplementary material for: Multidrug punch cards in primary care: a mixed methods study on patients' preferences and impact on adherence
Source: Front Pharmacol. 2014 Oct 1;5:220. doi: 10.3389/fphar.2014.00220 (PMC4181287; doi:10.3389/fphar.2014.00220)
Supplement: Supplementary file 1 [file DataSheet1.DOCX]

***Supplementary Material***

**Multidrug punch cards in primary care: a mixed methods study on patients’ preferences and impact on adherence**

**Fabienne Boeni^1^*, Kurt E. Hersberger^1^, Isabelle Arnet^1^**

^1^Pharmaceutical Care Research Group, Department of Pharmaceutical Sciences, University of Basel, Basel, Switzerland

*** Correspondence:**

Fabienne Boeni

Pharmaceutical Care Research Group

Department of Pharmaceutical Sciences

University of Basel

Klingelbergstrasse 50

CH-4056 Basel, Switzerland

fabienne.boeni@unibas.ch

1. **Supplementary Data**

German original transcribing of the quotations, divided into the sections of their appearance in the article.

## Reason to recommend multidrug punch cards

Difficulty/confusion: (P7) „Ich habe immer ein Durcheinander im Schrank xxx. Ich habe immer eine Kiste gehabt mit da einer Pille, da eine Pille. So verpackt [in üblicher Verpackung] oder? Dann habe ich einfach so kreuz und quer ‚tschak, tschak, tschak‘. Und mit der Zeit hat es mich gedünkt, es ist nicht gerade die beste Lösung, oder.“

Non-adherence: (P7) „Manchmal ist auch passiert, dass ich eine [Tablette] vergessen habe und so.“

(P2) „[…] entweder machen sie [die Apotheke] Päckchen wo nur 10 [Tabletten] drinnen sind, dann reicht es trotzdem nirgendswo hin. Oder die [die Apotheke] machen eine 50er oder 100er Packung [Tabletten] und die brauche ich auch nicht. Und dann geht viel verloren. Und so [mit der Wochenverpackung] habe ich wirklich nur die Medikamente, die ich brauche.“

## Advantages and disadvantages of multidrug punch cards

(P1) “Das ist wunderbar!”

(P1) „Das [die Wochenverpackung] vereinfacht mein Leben ungemein.“

(P5) „Schon wieder eine Sorge weniger für mich.“

(P8) Interviewer: „Und warum finden Sie es gut, wenn es [die Medikamente] so verpackt ist [in der Wochenverpackung]?“ - Patient: „Man hat eine Übersicht. […]“.

(P4) „Den Nachteil finde ich ein bisschen, dass man nicht über alle Tabletten eine Übersicht hat. Ich kann jetzt wirklich nicht… Da wo ein Herz drauf ist [auf der Tablette], da weiss ich es ist für das Herz irgendwie, aber im Grossen und Ganzen weiss ich nicht, was ich da [einnehme]… Gut, es steht alles hinten drauf, nicht, bei mir? Ich weiss nicht, ob sie das generell machen oder nicht?“

## Handling of the multidrug punch cards

(P8) „Denn ich muss sie bereit machen, sonst würde ich also wirklich… Das muss ich offen und ehrlich sagen, würde ich sie vergessen [die Tabletten].“

(P2) „[…]. Wenn ich natürlich weiss, ich gehe drei Tage weg, dann schneide ich ein Ding ab hier [von der Wochenverpackung].“

(P1) „[…]. Und danach habe ich einfach mal für heute Abend, damit ich nicht mehr lange muss ‚knüble‘, habe ich die zwei [Tabletten] wo ich sowie muss nehmen, habe ich hier hinein gedrückt [in die Wochenverpackung] und die Blaue [Tablette] habe ich mal schon heraus geschickt. So arbeite ich mit dem Blister [=Wochenverpackung].“

(P2) „Ja, würde sagen hat schon eine gewisse Sicherheit dahinter [der Wochenverpackung]. Dann bin ich sicher, ich habe das Richtige genommen, da.“

Group A/Knowledge: (P5) „Ich weiss genau was ich nehmen muss.“

Group A/Package insert: (P1) „Weil wenn ich muss Beipackzettel lesen, entweder ich muss oder ich will, danach habe ich das alles was da darauf steht. Und das will ich auf keinen Fall.”

B/Knowledge: (P9) Interviewer: "Wie gut wissen Sie Bescheid darüber, welche Tablette welche ist zum Beispiel?“ Patient: „Das weiss ich nicht.“

Group B/Package insert: (P8) Patient: „Aber was das andere ist, weiss ich nicht.“ - Interviewer: „Das wissen Sie nicht. Würden Sie es dann gerne wissen? Also stört Sie das, dass Sie es nicht wissen?“ - Patient: „Ja, ich weiss nicht, ob ich es gerade wissen möchte.“ - Interviewer: „Das heisst es passt Ihnen so?“ - Patient: „Wissen Sie, das würde mich… wenn wenn etwas ist, wo… Das tut mich dann so beschäftigen. […].“

Group B/Package insert/trust and fidelity: (P3) „Nein. Ich vertraue Ihnen, und den Ärzten. Das interessiert mich nicht, will, ich verstehe es ja sowieso nicht. Was drinnen ist und was drauf steht [in/auf der Wochenverpackung] und so weiter. Nein, schaue ich nie an.”

## Adherence

(P6) „Ja, ich halte mich an die Regeln. Dem wo ich vorgegeben bekomme jetzt von der Therapie soz… ja. Ich mache was ich sollte und nicht… Therapietreu, so halt. (Und bemüht?) Wenn man jetzt Medis bekommt zum sich beruhigen, wenn man eben… so Anfälle bekommt. Dann würde ich sagen, dann ist Therapietreue wenn man es wirklich nur dann nimmt.“

(P9) „Ich weiss es [die Medikamente] fängt viel ab, wenn man zwei Herzinfarkte hatte, danach weiss man, was es bedeutet, wenn man Medikamente nehmen muss. Dann nimmt man sie auch [die Medikamente].”

(P8) „Ja, was will ich? Es bleibt mir nichts anderes übrig. Es kommt ja nur mir zu Gute wenn ich es nehme, oder? Ich will da nicht noch einmal in das Spital hocken.“

(P10) „Wenn ich sie nicht nehmen würde, ich würde es spüren. Also ich müsste sehr wahrscheinlich bald einmal…, also vielleicht höchstens zweiten Tag [nach der verpassten Einnahme] müsste ich schon zum Arzt und sagen, es geht mir nicht mehr gut. Also ich würde es merken.”

(P5) „Es ist, es ist so automatisch, oder, wenn ich da sitze mein Morgenessen auf dem Tisch habe, dann mach ich nur so und dann hab ich‘s. Und dann mach ich es [die Medikamente] in den Teller und die Sache ist erledigt.“].

(P9) „Ich nehme immer alle. Ich nehme sie immer wie ich muss, da muss ich nicht kontrollieren.“

(P11) „Aber da hast du doch Kontrolle [mit der Wochenverpackung]! Da hast du, bist du sicher, dass du das Richtige [Medikament] genommen hast.“

(P2) „Ich sehe es auf den ersten Blick. Das habe ich gehabt, das habe ich genommen, das weiss ich.“
